# Effect of Fly-Ash Cenospheres on Properties of Clay-Ceramic Syntactic Foams

**DOI:** 10.3390/ma10070828

**Published:** 2017-07-19

**Authors:** Kristine Rugele, Dirk Lehmhus, Irina Hussainova, Julite Peculevica, Marks Lisnanskis, Andrei Shishkin

**Affiliations:** 1 Rudolfs Cimdins Riga Biomaterials Innovations and Development Centre of Riga Technical University (RTU), Institute of General Chemical Engineering, Faculty of Materials Science and Applied Chemistry, Riga Technical University, Pulka 3, LV-1007 Riga, Latvia; kristine.rugele@rtu.lv (K.R.); julitapeculevica@gmail.com (J.P.); 2 ISIS Sensorial Materials Scientific Centre, University of Bremen, D-28359 Bremen, Germany; dirk.lehmhus@uni-bremen.de; 3 MAPEX Center for Materials and Processes, University of Bremen, D-28359 Bremen, Germany; 4 Department of Mechanical and Industrial Engineering, Tallinn University of Technology, 19086 Tallinn, Estonia; irina.hussainova@ttu.ee; 5 SMW Engineering—Latvia, Kr. Barona 3, LV-1050, Riga, Latvia; ml@magnesium.com

**Keywords:** clay, illite clay, composite, ceramics, syntactic foam, fly ash cenosphere, mechanics of materials, mechanical properties, semi-dry formation

## Abstract

A low-density clay ceramic syntactic foam (CSF) composite material was successfully synthesized from illitic clay added by fly ash cenospheres (CS) using the semi-dry formation method. The content of CS varied in the range of 10, 30, 50 and 60 vol %. Furthermore, reference samples without cenospheres were produced for property comparison. The materials comprising different amount of the additives were fired at temperatures of 600, 950, 1000, 1050, 1100, 1150 and 1200 °C. Firing times were kept constant at 30 min. Processing characteristics of the materials were evaluated in terms of density achieved and shrinkage observed as functions of both the CS content and the sintering temperature. The compressive strength and water uptake were determined as application-oriented properties. Except for the reference and the low CS level samples, the materials show an increase in strength with the increase in firing temperature, and a decrease of mechanical reliability with a decrease in density, which is typical for porous materials. Exceptions are the samples with no or low (10 vol %) content of cenospheres. In this case, the maximum strength is obtained at an intermediate sintering temperature of 1100 °C. At a low density (1.10 and 1.25 g/cm^3^), the highest levels of strength are obtained after sintering at 1200 °C. For nominal porosity levels of 50 and 60 vol %, 41 and 26 MPa peak stresses, respectively, are recorded under compressive load.

## 1. Introduction

In the modern world, one of the most challenging issues is energy efficiency over the full life cycle of a product. For example, civil engineering and the construction industry aim at improvements in buildings’ energy balance, as the energy spent for the delivery of building materials to a construction site and a zero-waste closed loop material circular economy have now to be taken into account. Weight reduction, the increase of specific strength (MPa·kg^−1^·m^3^) and reuse of wastes can significantly contribute to solving these issues. One specific approach with regards to this is the utilisation of fly ash cenosphere (FAC) addition into bulks to lighten building materials without substantially compromising strength, through the development of clay-ceramic syntactic foams. FAC is a specific component of the fly ash emitted by coal combustion power plants and characterised by a low bulk density (0.2–0.8 g cm^−3^), excellent thermal (up to 1200 °C) and high chemical stability [1,2]. Recently, cenospheres have been used for the production of different types of syntactic foams with matrices, including a wide variety of polymers and metals [3,4,5,6,7,8]. The major advantage of cenospheres is their comparatively low cost, since the material is available as a by-product of coal-based energy generation. The mechanical properties of three-phase syntactic foams (SF) in terms of strength can theoretically exceed those of otherwise comparable two-phase foams thanks to the positive contribution of hollow filler particles’ shells, for example, towards mechanical performance [9,10]. These kinds of filler particles provide a low density and superior compressive strength, combined with enhanced acoustic and thermal damping characteristics [11,12,13]. In the last decade, the metal matrix SF added by the cenospheres have been studied intensively [7,14,15,16,17], alongside hybrid metal-matrix composites [18], advanced ceramic matrix materials [19,20,21], and concrete [22]. However, there is a lack of published information on natural clay ceramic matrix cenosphere-based SFs. In the present work, an alkali solution of NaOH 6 M was used to promote clay vitrification at a lower temperature than those applied in [23], and enhanced wetting between clay particles and cenospheres (CS). The study addresses development and characterisation of the low-density close porosity natural clay ceramics at a comparatively low processing temperature.

## 2. Materials and Methods

### 2.1. Materials

As a matrix material, natural homogenised illite red clay from Liepa deposition (*Lode* Jsc, Liepa, Latvia) was used. The CS (*Biotecha Latvia* Ltd, Riga, Latvia) were used as a low-density filler for the clay matrix. The homogenised clay was dried at 105 °C for 24 h, milled with a RETSCH PM 400 (*Retsch*, Haan, Germany) ball mill for 15 min, and sieved to limit the particle size to <100 µm. The chemical compositions of both the CS and the clay have been thoroughly studied previously and are specified elsewhere [18]. The granulometry was performed and the bulk density of CS grading composition was determined by the sieve analysis according to the standard DIN 1045-2 using an AS 200 digit (*Retsch*, Haan, Germany) vibratory sieve shaker device equipped with sieves with dimensions according to the standard EN 993-2. The grading composition was <63 µm is 1.70%, 63–75 µm is 3.86%, 75–150 is 94.30%, 600–1180 µm is 0.06% in mass percentage. The CS bulk density was defined by means of a Scott volumeter according to the ISO 3923-2-81 in 6 parallel measurements, and found to be 0.38 ± 0.0013 g cm^−3^.

The main constituents of both a space holder and a matrix were SiO_2_ and Al_2_O_3_, where the percentage of SiO_2_ was 53.8 ± 0.5 wt % compared to Al_2_O_3_ at 40.7 ± 0.7 wt % in the CS. In the clay, SiO_2_ content was slightly higher (62.5 ± 0.5 wt %), whereas the content of Al_2_O_3_ is 15.6 ± 0.7 wt %. Liepa clay also contained several distinctive components such as iron oxide (Fe_2_O_3_—6.88 ± 0.2 wt %), while other elements like Na, K, Ca, Mg, Ti (in the form of oxides) did not exceed 8 wt % in total [18]. As reported in [24,25], the phase composition of Liepa clay changes under thermal treatment. The initial illite phase becomes less pronounced at 950 °C and disappears at higher firing temperatures (up to 1200 °C). At the same time, the proportion of hematite increases, as well as that of the quartz phase.

### 2.2. Processing

The precisely weighed dry raw materials were mixed in a plastic container for 3 min in a 3-axis tumbler mixer, the *Inversina 2L* (*Inversina*, Wald, Switzerland), at a speed of 40 min^−1^. The mixture was then transferred into a stainless steel container with a rubber-coated anchor-type mixer, the *Clatronic Multi Food Processor KM3350* (*Clatronic* GmbH, Kempen, Germany)*,* at a rotation speed of 60 min^−1^. The components of the mixtures under consideration are listed in Table 1. Aqueous solutions of components 3–6 (Table 1) were slowly added into the mixer container and mixed for 5 min. For the components 1 and 2 (Table 1), both volume and mass percentages are given, while for components 3–6 only mass % is provided reflecting their use in the solution. The amount (by volume) of this mixture required to produce the cylindrical samples at an identical size (20.0 ± 0.5 mm in height) was empirically determined for each composition according to different bulk densities of the semi-dry mixture. The semi-dry clay-CS mixtures were pressed by uniaxial, double-side die-pressing in cylindrical templates of 20.0 mm diameter (applied force: 700 N; holding time: 40 s). Subsequently, the specimens were dried for 48 h at 22 °C in a desiccator above CaCl_2_ and NaOH (50 + 50% by mass). As a final stage, the materials were fired in a *NABERTHERM* Laboratory Furnace L9/13 with a P330 PID (*NABERTHERM*, Lilienthal, Germany) controller at temperatures of 600, 950, 1000, 1050, 1100, 1150 and 1200 °C, employing a heating rate of 5 K min^−1^ and a dwell time at the processing temperature of 30 min.

### 2.3. Composition

To prepare the materials of specified compositions, a 6 M NaOH solution (14 wt %) was used as a water and NaOH source for the clay green body formation promoting surface activation and vitrification. To facilitate sample compaction and compacted specimen extraction from the mould, the lubricant *Zusoplast G 72* (Zschimmer & Schwarz Gmbh & Co KG, Lahnstein, Germany) was used at a level of 2.0 wt %. Furthermore, the surfactant (−)-Ethyl L-lactate, (cleaning grade, ≥98.0%, *Sigma-Aldrich Chemie* Gmbh, Munich, Germany) was used to improve clay and CS particle wetting [18,24,26] at a level of 2 wt %. Another 0.5 wt % of a dispersing agent on huminite-silicate basis, *Dolaflux B 11* (Zschimmer & Schwarz Gmbh & Co KG, Lahnstein, Germany), was introduced. All the above weight percentage values reflect the dry clay-CS mixture as represented in Table 1, the clay/CS ratios also calculated in vol % for convenience. The specimens with 0, 10, 30, 50 and 60 vol % CS loading were designated as CE-0, CE-1, CE-3, CE-5 and CE-6, respectively.

### 2.4. Characterisation

Compression tests were conducted on an *Instron 8801 Universal Testing Machine* (Instron, Norwood, MA, USA) at room temperature and a constant quasi-static test speed of 3.0 mm min^−1^. The reported numbers represent the averaged values of at least 5 tested specimens of the same composition. An apparent density and porosity of the sintered syntactic foams were evaluated according to Archimedes’ principle [27]. The results were averaged over 7 samples per set of sintering conditions.

For microstructural characterization, a *Zeiss EVO MA-15* scanning electron microscope (SEM) (*Carl Zeiss AG*, Oberkochen, Germany) and Image Pro 7 image analysis tool by Media Cybernetics (version 7.0, Media Cybernetics, Inc., Rockville, MD, USA, 2011) were used. For the light microscopy (LM), a *Keyence VHX-2000* (Keyence Corp, Osaka, Japan) equipped with 54 Mpix Camera and lens VH-Z20R/Z20W (for ×20–200 times magnification), was utilised.

## 3. Results and Discussion

### 3.1. Appearance of Samples, Shrinkage

**Figure 1:**
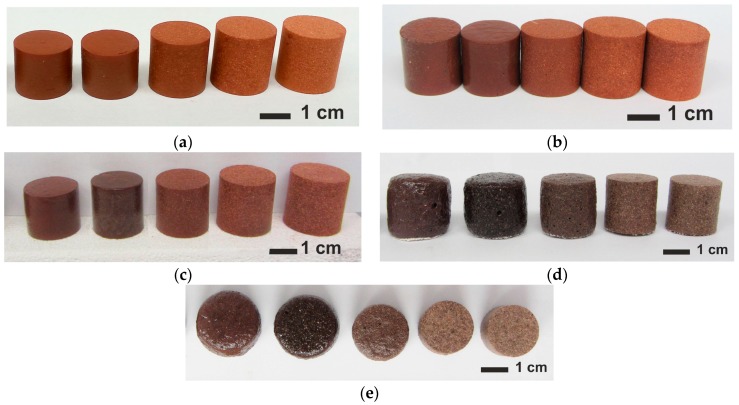
Appearance of samples with CS load (from left to right in each row) 0, 10, 30, 50 and 60 vol % of CS, (**a**) fired at 1000 °C, (**b**) 1050 °C, (**c**) 1100 °C, (**d**,**e**) 1200 °C—(**d**) side view and (**e**) top view.

**Figure 2:**
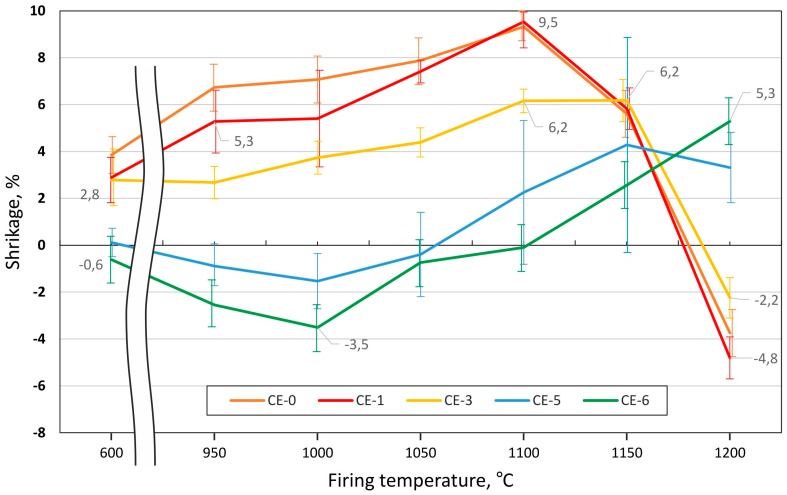
Dependence of total shrinkage on CS content and firing temperature.

Uniaxial die pressing yielded cylindrical ceramic syntactic foam (CSF) samples with an initial diameter of 20.0 ± 0.2 mm and a height of 20.0 ± 0.5 mm. Images of these samples after firing at different temperatures are collected in Figure 1. For firing temperatures from 600–1150 °C and all series, the exact cylindrical shapes expected, without visible deformation and cracks were obtained (Figure 1a–c). In contrast, the samples CE-0, CE-1 and CE-3 fired at 1200 °C exhibited some macroscopic distortion. The level of deformation decreased with increasing CS content (Figure 1d,e). Moreover, as shown in Figure 1a–c, the size of different specimens differs as a consequence of deviations in shrinkage induced by the different cenosphere content level. The dependence of shrinkage on CS content and firing temperature is represented in Figure 2. In the firing temperature range 600–1150 °C, the shrinkage decreases with increasing cenosphere content level, varying from 3.8–9.9% for CE-0 to −0.6–2.6% for CE-6, which corresponds to the sample appearance depicted in Figure 1a–c. Negative shrinkage corresponds to a specimens expansion. This is also confirmed by Figure 2. The cause of negative shrinkage, illite clay self-expansion, is explained by the clay melting and gashouse product realising [28,29].

### 3.2. Microstructure

**Figure 3:**
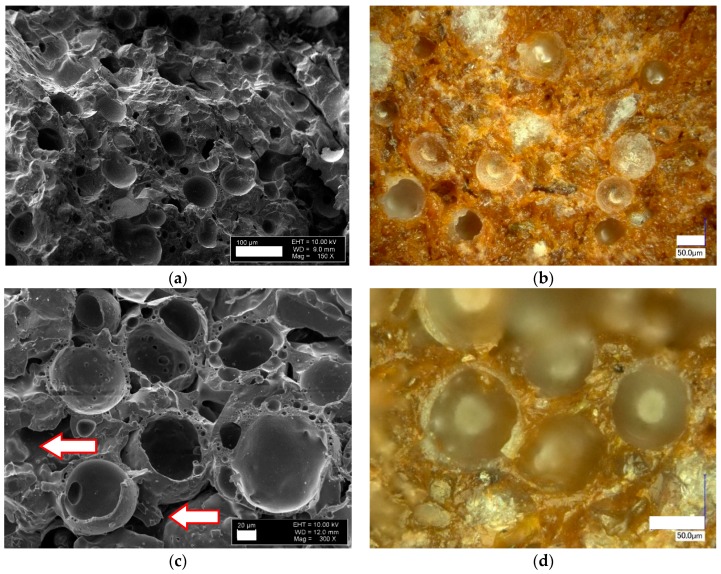

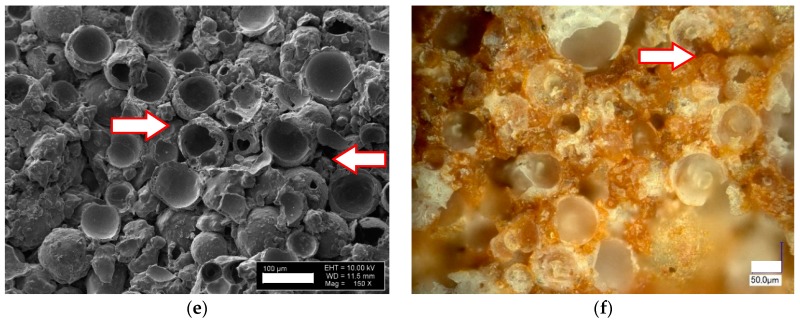
Fracture surfaces of fired samples with different CS loads, left, column showing scanning electron microscope (SEM), right, light microscopy images: (**a**,**b**) 10 vol % CS at firing temperature 1000 °C at a magnification of 150×; (**c**,**d**) 30 vol % CS at firing temperature 1100 °C at a magnification of 300×; (**e**,**f**) 50 vol % CS at firing temperature 1000 °C at a magnification of 150×.

The microstructure of the sintered samples is shown in Figure 3 for three different levels of CS content. The presence of three types of pores is clearly visible from Figure 3c. First, the CS internal void (size 50–100 µm); second, the closed pores in the CS walls (size 10 µm and less), and; third, the voids between CS (Figure 3c,e,f). For the latter, some further distinction is possible: samples processed at low firing temperatures show inter-particle open porosity. At higher firing temperatures, solid and liquid phase sintering leads to the closure of this type of porosity, while gas release from the matrix at firing temperatures exceeding 1100 °C results in closed porosity. The latter effect is most pronounced in samples containing low amounts of cenospheres. The same expression of porosity is characteristic for all series containing CS, with the exception of the reference series without CS only. 

In all cases the CS remain intact and are neither broken during the compaction, nor the sintering process. Due to the similarity between the chemical composition of CS and clay, contrast in SEM images is low, but light microscopy (LM) images allow distinguishing between the clay matrix material (brown colour) and the light grey colour of cenospheres. The brown colour of the clay is caused by the presence of Fe_2_O_3_ [18]. Optical images of samples with 10 and 30 vol % of CS addition (Figure 3b,d) demonstrate the integrity of the CS, as well as their even distribution in the clay ceramic matrix. Samples with 50 vol % of CS show inter-particle voids, but CS are still well surrounded by the clay matrix (Figure 3e,f).

**Figure 4:**
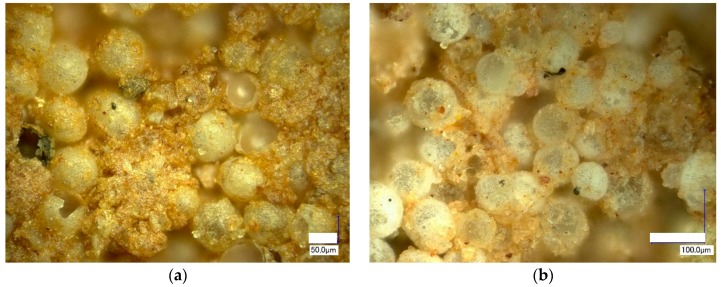

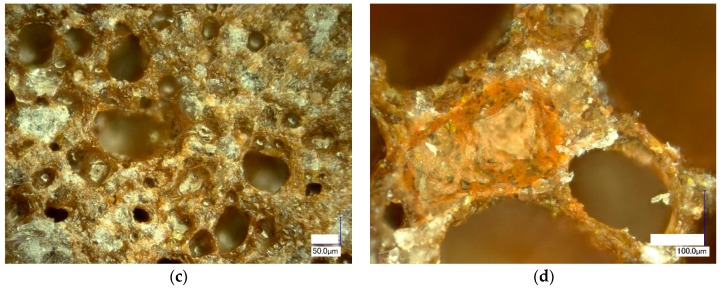
LM images (magnification 500×) of ceramic syntactic foam (CSF) samples (**a**,**b**) fired at 1000 °C with (**a**) 50 and (**b**) 60 vol % of CS addition (side view), (**c**,**d**) cross section of reference samples without CS addition fired (**c**) at 1150 and (**d**) 1200 °C.

During the illitic clay ceramic firing, two processes occur which lead to the samples’ changing dimensions. These are the positive shrinkage caused by ceramic particle reorganisation as a result of ceramic matrix densification and, at temperatures above 1150 °C (for some clays it could be 1100 or 1200 °C) [28,29], the aforementioned gas release, which coincides with the appearance of the liquid phase and thus leads to formation of bubbles, that is, the closed porosity in the matrix. In the latter case, the macroscopic effect is self-expansion (negative shrinkage) of the clay matrix. Figure 4 illustrates the phenomenon by clearly showing closed pores in the matrix in Figure 4c,d, which correspond to a firing temperature of 1150 °C (Figure 4c) and 1200 °C (Figure 4d), but not in Figure 4a,b, which reflect lower firing temperatures. 

As is demonstrated in Figure 3e,f and Figure 4a,b for series CE-5 and CE-6, the respective samples characteristically show high levels of open porosity caused by inter-particle voids. The phenomenon is described as channelization, as the resulting porosity provides paths that lead through the material. Via these channels, the gases which are typically generated in illitic clay at temperatures starting from 1150 °C [28,29] can escape. However, for samples CE-0, CE-1 and CE-3, the level of channelization is not sufficient for complete degassing.

**Figure 5:**
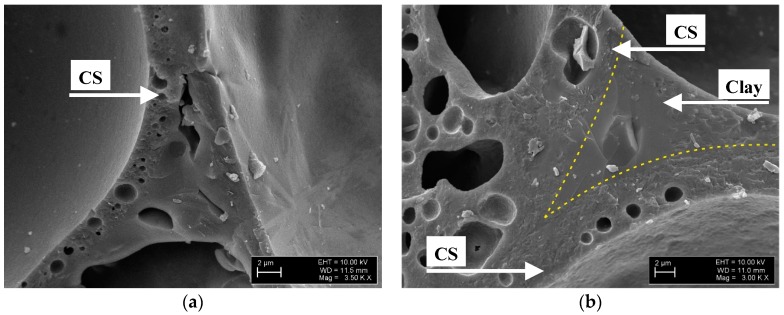
SEM images of CE-5 samples fracture fired at 1000 °C with 50 vol % of CS addition, (**a**) at a magnification of ×3500; (**b**) at a magnification of ×3000.

All samples demonstrate a strong integration of CS particles in the clay matrix (Figure 5a,b). In Figure 5b, the very good wetting between the CS particles and the clay ceramic matrix is clearly visible: the yellow dotted line in Figure 5b highlights the interface between the two components. Such beneficial interaction between the materials’ two major phases is effective already at firing temperatures of 1000 °C, as Figure 5a,b clearly show. However, at higher temperatures, the appearance of a liquid phase further promotes the formation of a stronger interface between cenospheres and the matrix. Specifically in comparison to the situation at very low firing temperatures, where a point-to-point contact (a) between matrix powder particles and cenospheres and (b) between the cenospheres themselves must be assumed, the partially liquid matrix in conjunction with the good wetting will significantly increase the matrix-cenosphere interface area and thus also strengthen contact points between individual cenospheres. Besides the area-based effect, geometrical aspects of the link between cenospheres will be improved, as once again Figure 5b illustrates: It shows that the kerf radius is significantly increased by the intermediary matrix phase. This effect will greatly reduce local, microscale stress concentrations, and thus contribute to the increase of macroscopic strength observed in samples containing 30, 50 and 60 vol % of cenospheres over the full range of firing temperatures covered, which is particularly pronounced from 1000 to 1200 °C. Even the later formation of pores in the matrix can be beneficial in this respect, as it creates an internal pressure in the matrix, which further supports contact and interface formation.

**Figure 6:**
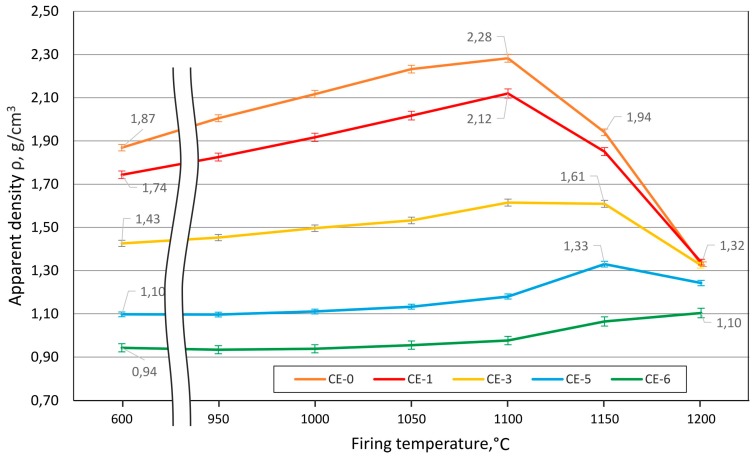
Apparent density dependence on the CS content and the firing temperature.

Samples with 50 and 60 vol % of CS addition (Figure 3e,f and Figure 4a,b) also show intact CS that is well distributed in the clay ceramic matrix, but in the case of samples with 60 vol % of CS addition the inter-particle voids decrease. Samples with 0 vol % fired at 1200 °C demonstrate negative shrinkage (self-expansion), which matches the appearance of bubbles of an approximate size of 20–150 µm in the matrix in Figure 4c. Samples with 0 vol % show the highest value of self-expansion—or minimal (negative) shrinkage—at −4.8% (fired at 1200 °C, Figure 2), which corresponds to a decrease of apparent density from 1.87 g/cm^3^ when fired at 600 °C to 1.32 g/cm^3^ when fired at 1200 °C, passing an intermediate maximum of 2.28 g/cm^3^ at 1100 °C (see Figure 6). At the same time, the images of samples shown in Figure 1d,e clearly illustrate the lowering of the amount of self-expansion with the increase in CS vol %. This observation is in direct correlation with the dependence of shrinkage on firing temperature (Figure 1 and Figure 2): at a firing temperature higher than 1100 °C and for samples with 0, 10 and 30 vol % of CS loading, the self-expansion process starts to dominate the evolution of density. Until then, a maximum of 9.5% expansion is reached for the CE-0 and CE-1 and 6.2% for the CE-3, which dramatically drops to negative values at 1200 °C (indicating shrinkage), with levels of −4.8%, −3.7% and −2.2%, respectively. In contrast, for compositions containing 50 and 60 vol % of CS content the sample volume continues to increase. For the samples with 60% CS volume content, it is significantly higher at 5.3% than for those with 50 vol % CS loading, which reach 3.3% expansion on average.

**Figure 7:**
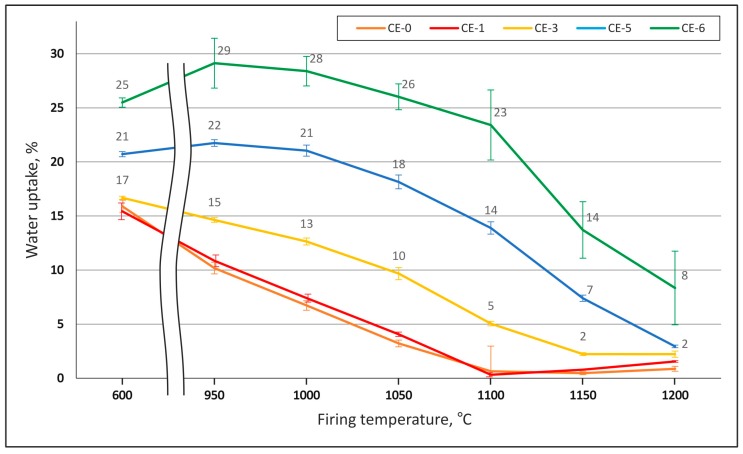
Dependence of water uptake on CS content and firing temperature.

This effect could be explained by the channelization effect caused by interparticle voids, which is supported by the microscopic images (Figure 2 and Figure 3). The phenomenon implies an increase in open porosity, which is reflected in the material’s water uptake. Figure 7 shows water uptake in relation to CS vol % content and firing temperature. The highest water uptake is observed for the series with 60 vol % of CS is 29% at a firing temperature of 950 °C. Also included in the diagram are graphs representing samples with 10, 30, 50 and 60 vol % CS. From series to series water uptake increases from an average of 3–4% at 600 °C to 5–7% at 950 °C and up to 8–7% at 1000 and 1050 °C. In parallel to the rising firing temperature, the difference between the series becomes more pronounced: at 1100 °C, we observed a 5% deviation between the CE-1 and CE-3 sample series, but already 10% between the CE-3 and CE-5 series. The smaller difference in microsphere content between the CE-5 and CE-6 series still leads to the 9% shift in water uptake. This tendency is continued at increasing firing temperatures up to 1200 °C, however, while the absolute difference increases, the relative change is even more pronounced: at 1150 °C, 2%, 5% and 7% change between the 10, 30, 50 and 60 vol % samples are found, while at 1200 °C, the respective values are 1, 1 and 5 vol %. Liquid phase and subsequent (closed) bubble formation are confirmed by the decrease in both apparent density and water uptake. All these changes are most apparent at the firing temperature range of 1100 to 1200 °C (see Figure 2, Figure 5, Figure 6 and Figure 7). The liquid phase present from 1100 °C upwards ensured good wetting between the matrix and the CS particles. The favourable wetting behaviour had already been observed by Shishkin et al. [18] and explained by the similarity of chemical and phase composition between Liepa clay and CS. The micrographs in Figure 3c,d and Figure 5a,b confirm this effect. The dotted line in Figure 5b highlights the transition from clay ceramic matrix to CS. 

### 3.3. Syntactic Foam Properties

#### 3.3.1. Density and Open vs. Closed Porosity Levels

Figure 6 below details the dependence of apparent density on CS content and firing temperature, while Figure 7 depicts the development of water uptake as a function of these two parameters. Similar to pycnometric data, water uptake can be interpreted as a measure of open porosity. In this respect, the general tendency of the reference material without cenospheres is also reflected in the cenosphere-containing variants: higher firing temperature results in lower water uptake.

When comparing the different curves rather than interpreting individual ones, the main difference observed between the reference and the syntactic foams is a general increase of water uptake with porosity, namely, cenosphere content. A possible cause of this is the hindrance towards densification which the cenospheres represent. This interpretation is supported by the fact that the higher cenosphere content levels approach the bulk density of the cenospheres themselves, which implies that, during firing, there are few remaining degrees of freedom for cenosphere movement and thus few possibilities for compensation of the density reduction experienced by the clay matrix. 

At first sight, this finding may seem to contradict Figure 6, which indicates a clear increase of porosity at firing temperatures above 1100 °C for samples containing lower amounts of cenospheres. However, since Figure 6 reflects total porosity, whereas Figure 7 only covers open porosity, the likely explanation is that the observed reduction in density is mostly based on closed porosity. This again matches the known process of gas evolution in the clay matrix, which leads to the development of closed porosity and has already been explained above in conjunction with Figure 3 and Figure 4. The fact that increasing the cenosphere content level means reduced clay matrix volume fraction also supports this interpretation, as it would imply that the density reduction effect caused by gas release and associated development of porosity in the matrix would diminish for syntactic foams with increasing filler-based porosity.

#### 3.3.2. Density and Porosity Levels vs. Compressive Strength

**Figure 8:**
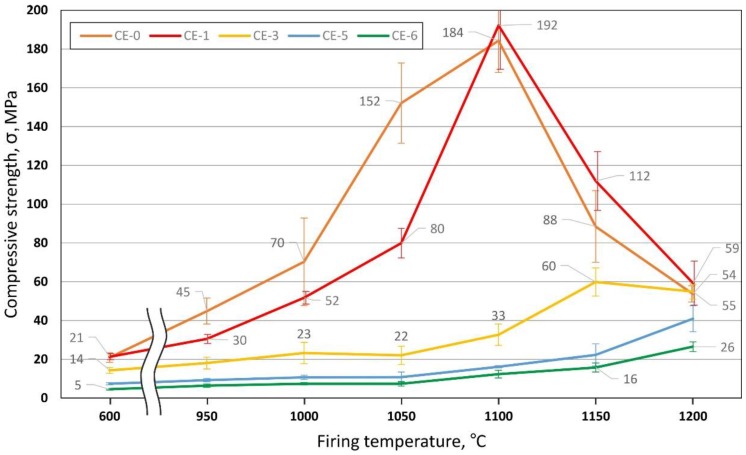
Compressive strength dependence on the CS content and the firing temperature.

Figure 8 provides an overview of the compressive strength of the material variants covered in the present study. As seen in exemplary stress-strain graphs, all material variants fail in a brittle manner, thus offering a clear indication of compressive strength as the stress value of the stress-strain curves’ first peak following the elastic region.

For almost any kind of foam, strength as well as Young’s modulus is directly linked to a relative density. Basic relationships in this respect have been proposed by Gibson and Ashby, with approximations assuming foam properties to vary proportionally with the value of relative density to the power of 1.5–2, depending on the characteristic in question [30]. Similar relationships have been proposed for syntactic foams, though in this case the drop in strength and stiffness caused which follows from density reduction is somewhat compensated by the supporting effect of the hollow filler particles’ shells, as exemplified in the equation below suggested by Wu et al. [7]:

σ_y_ = C (σ_m_ (1 − ϕ)^1.5^ + σ_fw_ϕ (1 − (1-t/R)^3^)^1.5^), (1)

In it, σ_y_ is the yield strength of the syntactic foam, while σ_m_ and σ_fw_ denote the yield strength of the matrix and the fracture strength of the hollow fillers’ wall. The volume fraction of spheres is denoted by the parameter ϕ, their wall thickness and radius by t and R, respectively. C is a general constant. For the present materials, both the Gibson-Ashby model and the syntactic foam one are relevant, because certain states of the foams discussed are essentially syntactic foams with a porous matrix.

Nevertheless, the development of strength as a function of firing temperature as depicted in Figure 8 basically captures the typical dependency when seen in conjunction with Figure 6. First, we have the apparent reduction in strength which results from cenosphere addition. Superimposed is the effect of matrix properties seen specifically in the peak associated with 0% and 10% of cenosphere addition at a firing temperature of 1100 °C, which marks the point of maximum apparent density, that is, lowest porosity, in the matrix. The subsequent increase in closed porosity in the clay matrix, which has been explained above, leads to a reduction of matrix strength once the threshold of 1100 °C firing temperature is exceeded. The deviating behaviour of syntactic foams containing higher amounts of cenospheres—these show maximum strength at firing temperatures of 1150–1200 °C—can be linked to an influence of the filler on the development of closed porosity at higher temperatures. Specifically, the presence of the filler in the syntactic foams will prevent the matrix from reaching densities near to the theoretical one prior to any gas evolution from the matrix. As a consequence, the inter-cenosphere matrix will still provide paths which allow gas release, thus impeding the formation of closed porosity observed in the reference samples. With this density-reducing mechanism rescinded, the appearance of a liquid phase at higher firing temperatures will instead facilitate rearrangement of cenospheres and thus density increase. Thus, effectively the syntactic foam matrix will densify at higher firing temperatures, which serves to explain both the overall density increase as well as the strength increase in the syntactic foam samples. 

At the same time, as explained in the previous section discussing the microstructural characteristics of the material, the appearance of a liquid phase will alleviate internal stress concentrations by introducing larger transition radii between neighbouring cenospheres. Macroscopically, this effect is reflected in higher strength levels.

Also at higher temperatures, a further phase formation reaction comes into effect: Wang et al. have described the formation of a mullite framework in comparable materials and under similar conditions [15]. As in the present case, they produced a cenosphere-based syntactic foam using a slurry prepared via ball-milling of fused silica powders (>99.9%). Their observations show that at least when using longer dwelling times of 120 min. at slightly lower firing temperatures, mullite crystals grow from the cenosphere shells into the matrix to finally form a mullite network, strengthening the matrix as well as the connection between matrix and filler. It is likely that despite the shorter dwelling times used in the present study, such phase formation will also take place in the materials investigated here. Arguments in this respect include the composition of Liepa illite clay with its SiO_2_ content of 62.55 ± 0.5 wt % as reported by Shishkin et al. [13], which is present in the form of quartz [22], as well as the higher firing temperatures. This may be expected to partly compensate shorter dwelling times also in view of the prolonged time associated with temperature ramp-up to the higher final values (216 min for heating up to 1100 °C; 226 min for reaching 1150 °C and 236 min for 1200 °C, each at 5 °C/min, starting from an initial 20 °C). Even if these conditions should not suffice to form a full network within the whole matrix, it would still strengthen the interface between the cenospheres as the origin of mullite crystal growth and the matrix.

**Figure 9:**
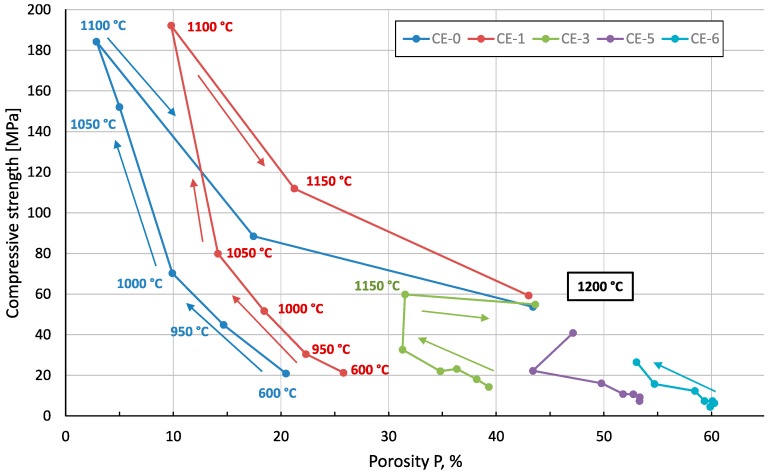
Dependency of compressive strength on porosity and firing temperature for material variants containing 0, 10, 30, 50 and 60 vol % of cenospheres. Arrows are meant to highlight the course of firing temperature increase for each curve.

Figure 9 further illustrates the interrelation between processing conditions, density and compressive strength. Specifically, for samples containing no or low levels of cenospheres (0 and 10 vol %), the message is that identical porosity levels result in different levels of strength depending on the firing temperature. CE-1 samples (10 vol % of cenospheres) fail at compressive stresses of less than 30 MPa when fired at 950 °C only, but can be loaded up to approximately 112 MPa when fired at 1150 °C, despite the fact that porosity is almost ideally matched in both cases. The explanation is the different character of the fraction of porosity not introduced by the cenospheres—in the low temperature case, it is predominantly open porosity, as can be deduced, for example, from Figure 4a,b, while firing at higher temperatures leads to closed porosity as exemplified in Figure 4c,d.

An interesting side aspect is the fact that strength, as well as porosity levels, of the sample series CE-0, CE-1 and CE-3 almost match for firing temperatures of 1200 °C, even though the origins and thus the nature of porosity should be different in all three cases: CE-3 contains mostly filler-based porosity, thus having basically a syntactic foam structure, and CE-0 contains only porosity originating from internal gas release in the liquid phase. CE-1 marks an intermediate position between these extremes. The conclusion to be drawn from this is that the present material appears not to experience any strengthening by the filler. This could be explained by the fact that other than in polymer matrix syntactic foams, in the present materials filler shell materials and matrix are both of a ceramic nature and show similar property values. However, the result may be seen to contradict findings on steel matrix syntactic foams, which show benefits in strength associated with the filler despite the fact that in some of these cases, both the Young’s modulus and the strength of the filler fell short of the matrix material properties [14,31,32]. Note, however, that in terms of filler wall strength this is not the case for syntactic foams using glass microspheres, for which strength values of up to 3500 MPa have been derived through simplified models of syntactic foam behaviour; the Young’s modulus and the strength of the filler fell short of the matrix material properties [14,32].

**Figure 10:**
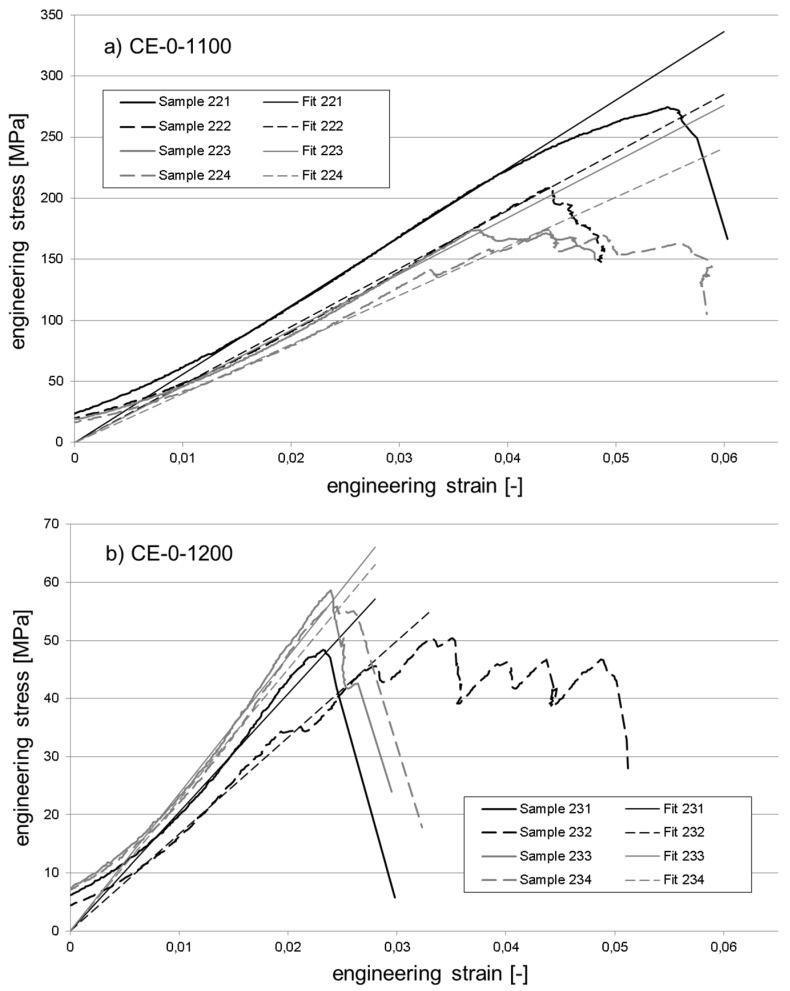
Exemplary stress strain curves for the reference material containing no cenospheres: (**a**) maximum strength at firing temperature 1100 °C; (**b**) strength and stiffness reduction at firing temperature 1200 °C.

**Figure 11:**
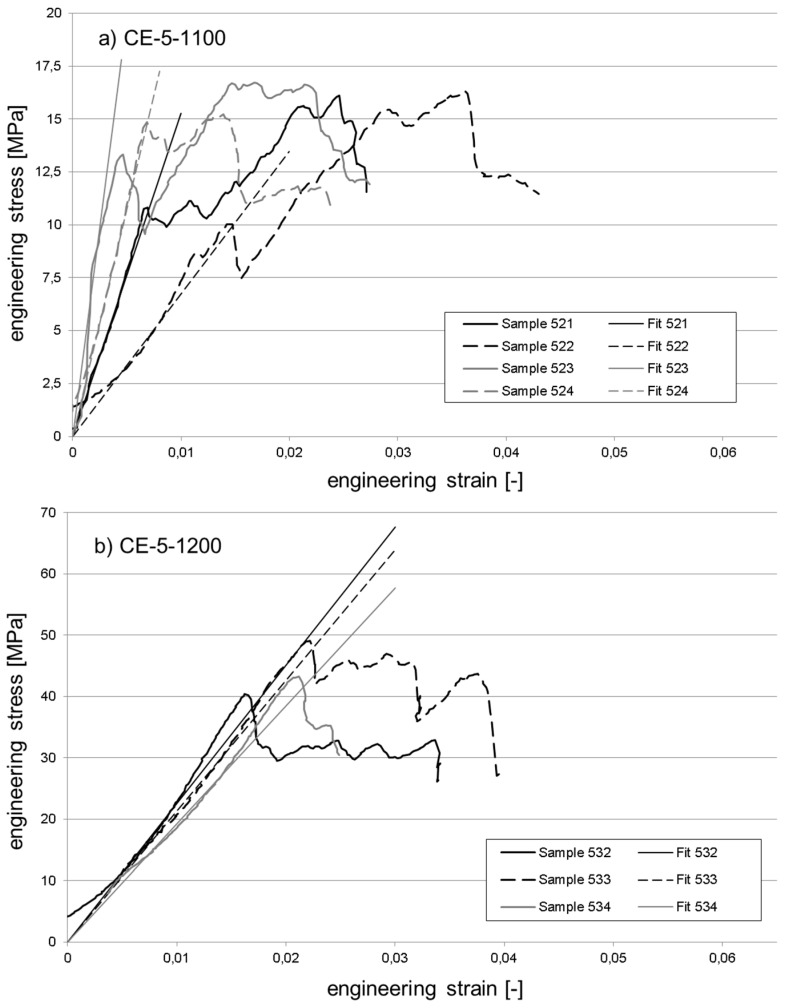
Exemplary stress strain curves for the syntactic foams containing 50 vol % cenospheres: (**a**) firing temperature 1100 °C; (**b**) firing temperature 1200 °C.

Figure 10 and Figure 11 exemplarily illustrate the compressive response of selected material variants. These are the reference material without cenosphere addition fired at 1100 °C (Figure 10a) and 1200 °C (Figure 10b), respectively, plus syntactic foams containing 50 vol % of cenospheres and once again fired at 1100 °C (Figure 11a) and 1200 °C (Figure 11b). The reader should note that while the strain range depicted is equal in all diagrams, the stress scale has been varied for reasons of clarity. All stress-strain curves have been modified by first adding a linear fit function determined for the central part of the elastic region. The curve intervals used as the basis of these fits were chosen to extend from stress levels corresponding to 25% and 75% of the respective sample’s strength value. The stress-strain as well as the fit curves themselves were then shifted along the *x*-axis in such a way that the latter passed through the origin in order to facilitate direct comparison of curves. 

When comparing the stress-strain curves across Figure 10 and Figure 11, some general differences become apparent. Almost irrespective of the firing temperature, the reference samples do not show an extended deformation range. Instead, in almost all cases, the main stress peak is followed by a steep drop in stress levels. Quite apart from that, the cenosphere-containing samples show a stronger tendency towards multiple stress peaks, which implies a more localized failure during which, for example, different cross sections fail consecutively, or breakup of the part occurs bit by bit, rather than via a single, complete disintegration event.

Approximations of the Young’s modulus as determined from the experimental curves in Figure 10 and Figure 11 in the manner described at the beginning of this section are summarized in Table 2. In general, the comparison reveals higher relative levels of scatter (as indicated by the value of the standard deviation estimator) for the syntactic foams, which is not in line with corresponding observations regarding compressive strength. Interestingly, in terms of the absolute values, the difference in Young’s modulus between samples fired at 1100 °C containing 0 and 50 vol % is slightly smaller than would have been expected from the difference in density and the general relationships between mechanical properties and density given further above. The respective syntactic foam samples have half the density of the reference material specimens (see Figure 6), but achieve 44% of their Young’s modulus. In contrast, when fired at 1200 °C, both materials show very similar densities and Young’s moduli.

## 4. Conclusions

The present study was dedicated to the evaluation of clay matrix syntactic foams using cenosphere-type hollow spheres as filler to introduce porosity. The work has succeeded in demonstrating the general viability of this approach, but it also indicated that the formation of the final foam is a complex process involving up to four different types of porosity, the development and expression of which is greatly influenced by the processing conditions applied. As a consequence, the compressive strengths of the materials produced do not show clear tendencies, such as, for example, a monotonous dependence of compressive strength on cenosphere content level and/or firing temperature. To provide an example, in terms of compressive strength, the optimum firing temperature turns out to be 1200 °C for samples containing 50 and more vol % of microspheres, while it is 1100 °C for samples with cenosphere volume percentages of and below 10%.

It appears that, for the higher porosity materials, there is no specific, direct strengthening effect of the cenospheres, which differs, for example, from our experience with metal matrices and specifically with polymer matrix syntactic foams. The interpretation of these results is made more complex by the fact that specifically in the syntactic foams we have to take into account the aforementioned, different types of porosity over the firing temperature range (open-cell porosity in matrix at lower firing temperatures, closed-cell porosity in matrix at higher firing temperatures, filler-based porosity independent of the firing temperature). The effect as such is visible through trends in density, water uptake and pycnometric measurements.

However, the lack of a pronounced increase of strength associated with the structural characteristics of a syntactic foam (as exemplified by Equation (1) above through the addition to strength of the hollow particle shell material) may partially be explained by the similarity in composition and structure between cenosphere shell and matrix material: basically, this similarity shifts the material towards a two-phase foam, whereas common syntactic foams profit from their three phase nature. In polymer matrix foams, the shell materials typically outperform the matrix in terms of mechanical characteristics, an effect which overcompensates the slightly higher density of the shell material. In contrast, in steel matrix syntactic foams, the absolute strength and stiffness of the matrix material exceed the corresponding characteristics of the shell material, however, the lower density of the latter allows it to surpass the weight-specific characteristics of the matrix. Thus in both cases, the syntactic foams directly profit in some way or another from their three-phase nature. This advantage is lost in the case of the present foams, where the hollow filler mainly allows porosity to be introduced in a more controlled manner than that achievable through stochastic effects like bubble formation based on internal gas release. Still, the increase in strength of medium-to-high porosity syntactic foams with firing temperature is an interesting phenomenon which deserves additional attention in the course of future work in the field. At present, it can qualitatively be explained by assuming three parallel effects:

- Formation of improved interfaces between cenospheres and matrix and specifically between cenospheres (in this case via the matrix), with the increase of interface area as the main aspect.
- Reduction of internal kerf radii and thus of local stress concentration, with the elimination of potential failure initiation sites as a consequence.
- Formation of mullite crystals originating from the cenospheres and growing into the matrix, with the possible formation of a full mullite framework within the matrix, and as a result strengthening of (a) the cenosphere-matrix interface and (b, potentially) the matrix itself.

## Figures and Tables

**Table 1:** Composition and designation of samples.

Nr	Comp.	wt %	g·cm^−3^	CE-0		CE-1		CE-3		CE-5		CE-6	
				vol %	wt %	vol %	wt %	vol %	wt %	vol %	wt %	vol %	wt %
1	Clay	-	2.2 ^1^	100.0	84.4	90.0	81.4	70.0	73.8	50.0	63.2	40.0	56.1
2	CS	-	0.74 ^1^	0	0	10.0	3.0	30.0	10.6	50.0	21.2	60.0	28.3
3	NaOH 6M	14	1.22 ^2^	-	11.8	-	11.8	-	11.8	-	11.8	-	11.8
4	Zusoplast G 72	2	1.10 ^2^	-	1.69	-	1.69	-	1.69	-	1.69	-	1.69
5	Surfactant	2	1.03 ^2^	-	1.69	-	1.69	-	1.69	-	1.69	-	1.69
6	Dolaflux B 11	0.5	0.80 ^3^	-	0.42	-	0.42	-	0.42	-	0.42	-	0.42

^1^ True, particle-based density, i.e., average density of a single, intact particle (shell + inner void). ^2^ Specific gravity of the liquid/solution. ^3^ Bulk density. Cenospheres (CS).

**Table 2:** Overview of Young’s modulus values obtained from the stress-strain curves exhibited in Figure 10 and Figure 11.

Designation		CE-0-1100	CE-0-1200	CE-5-1100	CE-5-1200
CS Content (vol %)		0	0	50	50
Firing Temp. (°C)		1100	1200	1100	1200
Tangent Modulus (MPa)	no. of samples	4	4	4	3
	min. value	4019.2	1664.2	673.4	1109,8
	max. value	5611.4	2359.2	3957.1	2254,5
	average	4744.9	2079.9	2078.7	1855
	standard deviation	657.9	306.7	1392.2	515,4
